# High Throughput Preparation of Aligned Nanofibers Using an Improved Bubble-Electrospinning

**DOI:** 10.3390/polym9120658

**Published:** 2017-11-29

**Authors:** Liang Yu, Zhongbiao Shao, Lan Xu, Mingdi Wang

**Affiliations:** 1 National Engineering Laboratory for Modern Silk, College of Textile and Engineering, Soochow University, 199 Ren-ai Road, Suzhou 215123, China; yuliangsuda@163.com (L.Y.); sdszb2015@163.com (Z.S.); 2 School of Mechanical and Electric Engineering, Soochow University, 178 Ganjiang Road, Suzhou 215021, China

**Keywords:** bubble-electrospinning, aligned nanofibers, high throughput, properties

## Abstract

An improved bubble-electrospinning, consisting of a cone shaped air nozzle, a copper solution reservoir connected directly to the power generator, and a high speed rotating copper wire drum as a collector, was presented successfully to obtain high throughput preparation of aligned nanofibers. The influences of drum rotation speed on morphology and properties of obtained nanofibers were explored and researched. The results showed that the alignment degree, diameter distribution, and properties of nanofibers were improved with the increase of the drum rotation speed.

## 1. Introduction

Electrospinning provides a simple and convenient method for generating nanofibers. Many outstanding characteristics of nanofibers, such as high ratio of surface area and superior thermal and mechanical properties, lead to their wide application in the fields of filtration, tissue engineering, drug delivery, health protection, and so on [1,2,3,4,5,6]. Many efforts have concentrated on enhancing the production of nanofibers to broaden their applications [7,8,9,10]. 

Bubble-electrospinning (BE) is one of the most influential methods of needleless electrospinning, and it provides a high-throughput method to produce nanofibers [11]. The BE setup consists of a slim metal electrode that is connected to the power generator and introduced inside a polymer solution reservoir, along with a thin gas tube which goes through the reservoir from its bottom to produce bubbles. The metal electrode leads to lower applied voltage which results in relatively low production of nanofibers. Therefore, a modified free surface electrospinning (MFSE), based on BE, is presented to enhance the production of quality nanofibers in our previous work [10]. The MFSE setup consists of a copper solution reservoir which is directly connected to the power generator, and a cone shaped air nozzle combined with the solution reservoir to generate bubbles. The MFSE setup can be applied with a much higher voltage than the BE setup. It is well known that applied voltage is a very important parameter influencing the formation of nanofibers, and higher applied voltage will result in improving the diameter distribution, decreasing the nanofiber diameter and enhancing the nanofiber throughput [10,12]. Therefore, high throughput fabrication of quality nanofibers can be easily obtained by MFSE under a much higher applied voltage.

**Figure 1:**
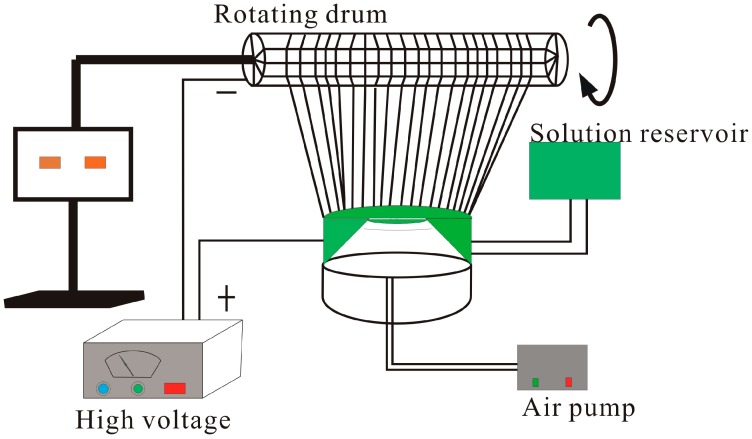
Schematic of the improved BE apparatus.

However, the nanofibers collected in the MFSE process are randomly oriented and are in the form of a nonwoven mat. Compared with disorderly nanofibers, the highly aligned nanofibers have better mechanical properties and biocompatibility. Therefore, it is desirable to generate high-throughput aligned nanofibers to broaden the application prospect in the fields of optoelectronic devices and biomaterials [13,14,15,16]. In this study, an improved BE (IBE), using MFSE combined with a high speed rotating copper wire drum as a collector, was presented successfully to obtain high throughput preparation of aligned nanofibers. Figure 1 showed the schematic presentation of the IBE setup. The effects of drum rotation speed on morphology and properties of obtained nanofibers were investigated by means of scanning electron microscopy (SEM), universal testing machine, X-ray diffraction (XRD), and other methods. The results showed that high throughput preparation of aligned nanofibers was successfully obtained by IBE, and with the increase of the drum rotation speed the alignment degree and diameter distribution of aligned nanofibers were improved, and mechanical and hydrophobic properties of the nanofibers were also enhanced.

## 2. Materials and Methods 

### 2.1. Materials 

Polyacrylonitrile (PAN, *M*_W_ = 150,000) powder, was purchased from Beijing Lark Branch Co., Ltd. (Beijing, China). *N*,*N*-dimethylformamide (DMF) was provided from Shanghai Chemical Reagent Co., Ltd. (Shanghai, China). All materials were of analytical grade and applied without any further purification.

### 2.2. Fabrication of Highly Aligned PAN Nanofibers by the IBE

The effects of electrospinning parameters—such as collecting distance, applied voltage, polymer concentration, and solvent—on the morphologies of electrospun nanofibers and their dimensions have been fully researched in [12,17,18,19,20,21] and our previous works [10,22,23]. Yordem [17], Yang [18], and Kong et al. [19] studied experimentally and theoretically the influence of collecting distance on the morphology and diameter of nanofibers. The results showed that when the collecting distance was too small, the nanofibers were a lot of beads due to insufficient solvent evaporation and nanofibers which were not fully stretched. With the increase of collecting distance, the charged jet could be accelerated to a higher velocity before it was collected, leading to the disappearance of beads and a decrease in nanofiber diameter. However, when the collecting distance was too big the electric field intensity would be smaller, resulting in low nanofiber production and increasing nanofiber diameter. Sao [10] and Qin et al. [12] found higher applied voltage could decrease the nanofiber diameter and improve the nanofiber production. Zhang [20] and He et al. [21,22] investigated the effects of polymer concentration and solvent on the morphology of nanofibers. The results illustrated that, with the increase of polymer concentration, the morphologies of nanofibers varied from a few beads, to smooth surfaces, then finally to nanofibers with larger diameter. There were possible factors affecting the nanofiber morphologies in different solvents, such as solvent volatility, solvent polarity, solution conductivity, and surface tension. The optimal choice of the solvent was that the electrospinnablility and efficiency were better, leading to decreasing and even eliminating the by-products in spinning process. In addition, Zhao et al. [23] researched the influence of solvent volatilization on the morphology and diameter of nanofibers, and exhibited with the increase of high volatility solvent in mixed solvent system, the beads gradually reduced and the nanofiber surface changed from roughness to formation of the pores. Moreover, with further increased high volatility solvent, evident pores and increasing nanofiber diameters appeared.

As discussed above, the IBE solution was obtained by dissolving 8 wt % of PAN in DMF under magnetic stirring for 2 h at 60 °C until it became homogeneous. The IBE experiments were carried out at room temperature (25 ± 2 °C) and a relative humidity of 55%. The applied spinning voltage was 45 kV, and the distance from the surface of the solution to the rotating drum was 18 cm. The drum rotation speed varied from 0 to 1200 r/min.

### 2.3. Measurements and Characterizations

The morphologies of aligned PAN nanofibers were observed by a scanning electron microscopy (SEM, Hitachi S-4800, Tokyo, Japan) at an acceleration voltage of 3 kV. The matrix morphology and nanofiber diameter distribution were obtained using Image J software (National Institute of Mental Health, Bethesda, MD, USA). The mechanical properties of PAN nanofiber membranes were characterized by a universal electromechanical test machine Instron-3365 (Instron Corporation, Norwood, MA, USA). The X-ray diffraction (XRD) patterns of PAN nanofiber membranes were recorded by Philips X’Pert-Pro MPD (PANalytical, Almelo, The Netherlands) equipped with Cu-Kα irradiation at a scan rate of 2°/min, and the crystallinities were calculated using Jade software (Java Agent Development Framework, Jade Software Corporation, Sydney, Australia). The wetting property and static contact angle (CA) of PAN nanofiber membranes were investigated by a Krüss DSA 100 apparatus (Krüss Company, Hamburg, Germany).

## 3. Results and Discussion

### 3.1. Morphological Characterization of Highly Aligned PAN Nanofibers (SEM)

**Figure 2:**
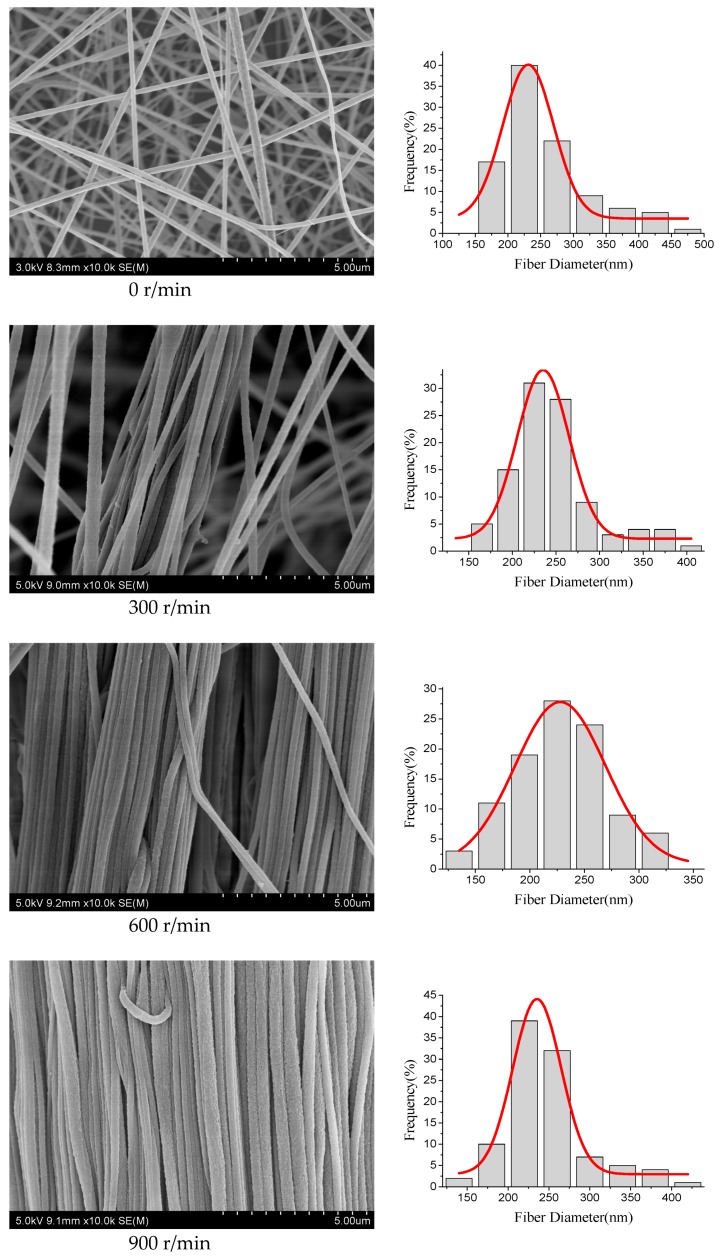

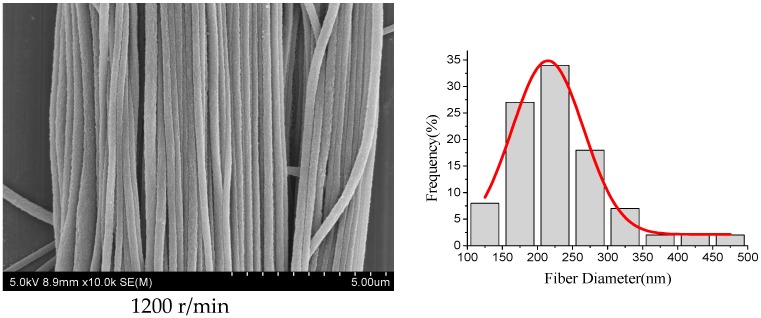
SEM pictures of the highly aligned PAN nanofibers with the different drum rotation speed. The right figures were the according diameter distribution.

**Figure 3:**
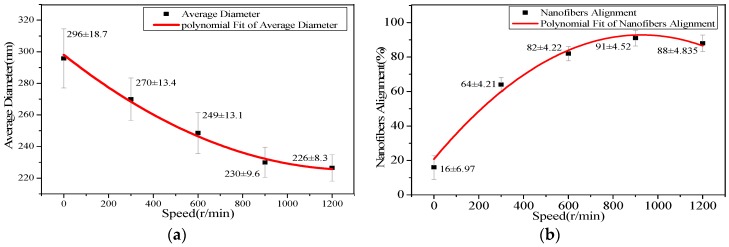
Effects of the drum rotation speed on average diameter and alignment of PAN nanofibers. (**a**) Average diameter; (**b**) nanofiber alignment.

The surface morphologies of highly aligned PAN nanofibers were characterized by SEM. Diameter distribution and average diameter of these nanofibers were determined by measuring 100 nanofibers selected randomly from the SEM pictures using image J software. Figure 2 illustrated SEM pictures of the highly aligned nanofibers obtained using controlled rotation speed of the drum, and the right figures were the according nanofiber diameter distribution. It could be seen that with the increase of the drum rotation speed the diameters of nanofibers decreased, and the degree of alignment of the nanofibers firstly increased rapidly and then tended to be stable. The relationship between the drum rotation speed and the average diameter of nanofibers was shown, respectively, in Table 1 and Figure 3a. The average diameter of nanofibers continuously decreased from 296 to 226 nm as the speed of the rotating drum increased from 0 to 1200 r/min. This decrease of diameter with increase of rotation speed might be due to the drafting force of the rotating drum. This drafting force not only caused nanofiber stretching and decreased the diameter, but also aligned the nanofibers.

In order to calculate the alignment degree, the angles (θ) between the long axial orientation of the nanofibers and their subject direction were used as the parameters to determine the alignment. The alignment degree of nanofibers was defined as the ratio of the number of nanofibers, whose angle values were between −5° and 5°, to the total number of all nanofibers. Figure 3b displayed the effects of the drum rotation speed on the alignment degree of PAN nanofibers. When the drum rotation speed was 0 r/min, the nanofibers were deposited randomly. When the drum rotation speed was 300 r/min the nanofibers started to become aligned. As the speed of rotating drum increased from 300 to 1200 r/min, the alignment degrees of nanofibers increased, and they were respectively 64, 82, 91, and 88%. The alignment of nanofibers might be due to the fact the nanofibers were mechanically pulled and stretched. In addition, the matching of velocities of the jets and drum rotation could result in obtaining highly aligned nanofibers due to the increased jet stability. Therefore, the drum rotation speed was an important parameter, which could control the diameter and alignment degree of nanofibers.

### 3.2. Mechanical Properties Analysis (Universal Testing Machine)

**Figure 4:**
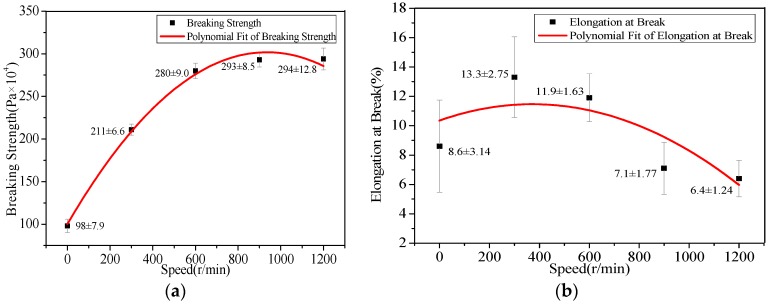
Mechanical properties of aligned PAN nanofiber membranes with the different rotation speed of drum. (**a**) Breaking strength; (**b**) elongation at break.

The effects of the drum rotation speed on the mechanical properties, such as elongation at break and tensile strength of random and aligned PAN nanofiber membranes, were presented in Table 2 and Figure 4. It was seen that with the increase of drum rotation speed, the tensile strength of nanofibers increased initially and then tend to stabilize, and the elongation at break of nanofibers increased firstly and then decreased. Compared with the random nanofiber membranes, the mechanical properties of aligned nanofiber membranes were enhanced. This means that the nanofiber alignment has a profound effect on the mechanical properties of nanofibers, especially tensile strength. However, when the drum rotation speed was increased to 900 r/min, the elongation at break of aligned nanofibers decreased, which could be the result of the excessive drafting force due to the excessive rotation speed of the drum.

### 3.3. Wetting Properties

**Figure 5:**
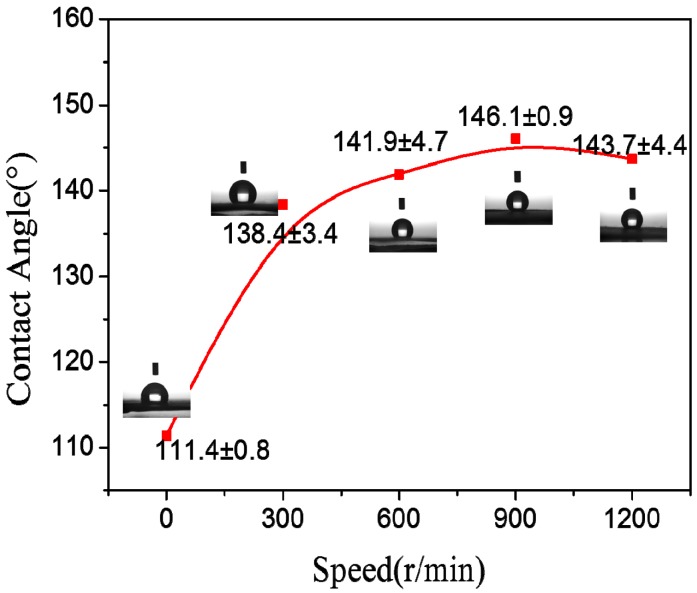
The effects of the drum rotation speed on the CA of PAN nanofiber membranes.

Figure 5 showed the effects of the drum rotation speed on the static contact angle (CA) values of electrospun PAN nanofiber membranes. It could be seen that the membranes were hydrophobic materials, and with the increase of the drum rotation speed, the CA increased and the hydrophobicity became stronger. That meant the nanofiber alignment could enhance the hydrophobicity of nanofiber membranes. It was reported that the nanofiber alignment could make hydrophobic materials more hydrophobic [15]. 

### 3.4. X-ray Diffraction (XRD) Spectrum Analysis

**Figure 6:**
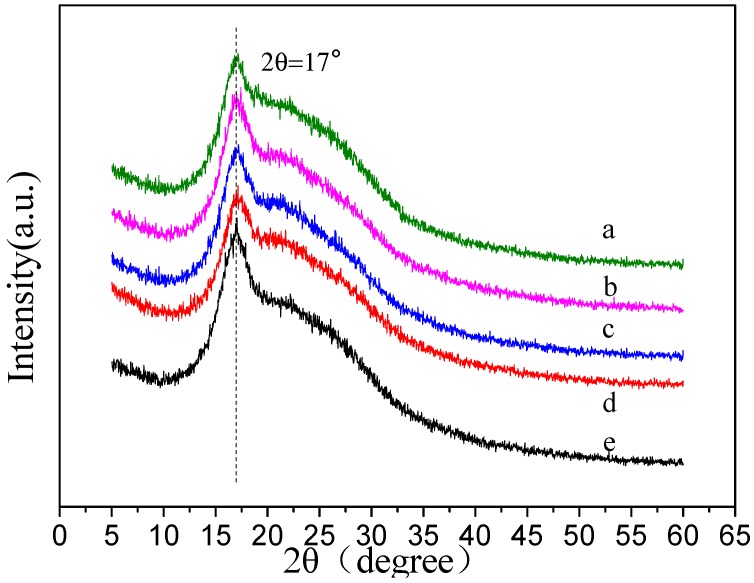
XRD spectra of PAN nanofibers under the different drum rotation speed, (**a**) 0 r/min; (**b**) 300 r/min; (**c**) 600 r/min; (**d**) 900 r/min; (**e**) 1200 r/min.

In order to illustrate the effects of the drum rotation speed on the crystalline structure of random and aligned PAN nanofibers, their XRD patterns with distinctive crystalline peaks were presented in Figure 6. It could be observed that the PAN nanofibers showed a strong diffraction peak at 2θ = 17°, which could be assigned as (200) crystal planes of PAN [2]. The XRD results exhibited that PAN nanofiber membranes still retained their crystalline structures with the increase of the drum rotation speed, and there was no new crystalline phase. The crystallinities of the five membranes were calculated by separating intensities due to amorphous and crystalline phase on diffraction phase, and the total area of the diffracted pattern could be divided into amorphous area ($A_{a}$) and crystalline area ($A_{c}$) [2]. The crystallinity, $X_{c}$, would be calculated by the relation $X_{c}=\frac{A_{c}}{A_{c}+A_{a}}\times 100\%$, and the results were respectively 28.0, 34.8, 35.5, 35.9, and 40.9%. It could be seen that with the increase of the drum rotation speed, the crystallinities of PAN nanofibers increased. It was possible that the nanofibers were mechanically pulled and stretched by the high speed rotating drum, leading to the higher crystallinity.

## 4. Conclusions

In this paper, an improved BE, consisting of a cone shaped air nozzle, a solution reservoir made of copper tubes, and a high speed rotating copper wire drum as a collector, was presented successfully to obtain high throughput preparation of aligned PAN nanofibers. The effects of drum rotation speed on morphology and properties of obtained nanofibers—such as the alignment degree, diameter distribution, mechanical property, wetting property, and crystallinity of nanofiber membranes—were investigated by scanning electron microscopy (SEM), universal testing machine, X-ray diffraction (XRD), and other methods. 

SEM images illustrated that with the increase of the drum rotation speed, the average diameters of the nanofibers decreased and the alignment degree was improved. The measurement of wetting properties and mechanical properties exhibited that the tensile strength and hydrophobicity of aligned PAN nanofibers were obviously enhanced compared with those of random PAN nanofibers. These results showed that the morphology and properties of PAN nanofibers were improved with the increase of the drum rotation speed.

## Figures and Tables

**Table 1:** The relationship between the drum rotation speed and the average diameter of nanofibers.

Rotating speed (r/min)	Average diameter ($\bar{D}$) (nm)	Standard deviation (*σ*) (nm)	Confidence interval (nm)
0	296	95.5	±18.7
300	270	68.3	±13.4
600	249	67.0	±13.1
900	230	49.0	±9.6
1200	226	42.6	±8.3

**Table 2:** The relationship between the drum rotation speed and the tensile strength/elongation at break of aligned nanofibers.

Rotating speed (r/min)	Tensile strength (Pa × 10^4^)	Elongation at break (%)
0	98 ± 7.9	8.6 ± 3.14
300	211 ± 6.6	13.3 ± 2.75
600	280 ± 9.0	11.9 ± 1.63
900	293 ± 8.5	7.1 ± 1.77
1200	294 ± 12.8	6.4 ± 1.24
